# Comparison between home-based and supervised rehabilitation protocols after anterior cruciate ligament reconstruction: a systematic review and meta-analysis

**DOI:** 10.1530/EOR-2024-0216

**Published:** 2025-09-04

**Authors:** Waleed D Khubzan, Khalid M Alhomayani

**Affiliations:** Department of Surgery, College of Medicine, Taif University, Taif, Kingdom of Saudi-Arabia

**Keywords:** anterior cruciate ligament reconstruction, home-based rehabilitation, supervised rehabilitation, functional recovery, systematic review

## Abstract

**Purpose:**

- This systematic review and meta-analysis assesses the comparative effectiveness of home-based and supervised rehabilitation protocols following anterior cruciate ligament reconstruction, with a focus on functional recovery and muscle strength.

**Methods:**

- Registered in PROSPERO (CRD42024585478) and following PRISMA guidelines, we searched Web of Science, PubMed, Ovid MEDLINE, and Cochrane. For the quality assessment, the Cochrane Collaboration risk of bias (RoB) tool was used. Review Manager v5.4 was used for the analysis.

**Results:**

- Twelve studies were included qualitatively, and seven quantitatively. Outcomes included subjective knee scores (Lysholm, Tegner, IKDC) and quadriceps/hamstring strength measures. Our findings indicated a 19% improvement in subjective knee scores for home-based protocols in some studies. However, for hamstring strength measures, the supervised protocol was superior (SMD = −0.48, *P* = 0.02).

**Conclusion:**

- Overall, home-based rehabilitation outcomes were comparable to supervised programs in functional recovery. However, supervised approaches slightly enhanced muscle strength. Supervised rehabilitation is recommended to optimize strength, especially for athletes, though further research is needed to assess its impact on return to sport.

## Introduction

Anterior cruciate ligament (ACL) is one of the most frequently injured structures in the knee, with an estimated 100,000–200,000 cases annually in the U.S (1, 2). Recent studies found that approximately 7% (6.9–7.5%) of athletes experience an ACL tear, with higher incidence among female athletes (8.9%) (3, 4). Another study conducted in Australia over 20 years found an annual growth rate of 10% in the incidence of ACL injury (5). Due to its limited healing capacity, surgical intervention, specifically ACL reconstruction (ACLR) using autologous or allogenic grafts, is often necessary to restore knee stability and function (2, 6, 7). Indications for ACLR include knee instability and the desire to resume physical activity or sports (8). While ACLR is typically successful, inadequate postoperative therapy can result in re-injury and diminished performance (9, 10, 11, 12), making structured rehabilitation essential for recovery (13) and aiming to restore knee function, improve proprioception, and support the return to sports (14). Traditionally, supervised rehabilitation has been the standard approach, but home-based rehabilitation (HBR) programs are gaining popularity for their convenience and cost-effectiveness (15, 16, 17, 18). A meta-analysis compared supervised to less supervised rehabilitation following ACLR and found no significant difference between the two approaches (19). Another recent meta-analysis found no significant difference between supervised and unsupervised rehabilitation after knee arthroplasty (20). Uchino *et al.* once again found no difference between both approaches following ACLR (21). While these studies suggest that the level of supervision may not significantly impact rehabilitation outcomes, a comprehensive and up-to-date systematic review specifically and directly comparing home-based and supervised rehabilitation programs across procedures is still lacking.

This systematic review aims to fill that gap by evaluating and comparing the outcomes of both rehabilitation approaches after ACL reconstruction, thereby informing clinical decision-making and improving patient care.

## Methods

### Study design

This systematic review and meta-analysis were conducted and reported in accordance with the Preferred Reporting Items for Systematic Reviews and Meta-Analyses (PRISMA) guidelines. The protocol was registered in the International Prospective Register of Systematic Reviews (PROSPERO: CRD42024585478) and met all the eligibility criteria for protocol registration.

### Objective

This study aims to systematically evaluate and compare the clinical effectiveness of HBR protocols versus supervised rehabilitation programs in patients undergoing ACL reconstruction, focusing on outcomes such as subjective knee scores, functional knee scores, quadriceps and hamstring strength measures.

### PICO framework

Population (P): patients who have undergone ACL reconstruction.

Intervention (I): HBR protocol.

Comparison (C): supervised rehabilitation protocol.

Outcomes (O): subjective and functional knee outcomes assessed using the Lysholm Score, Tegner Activity Scale (TAS), and the International Knee Documentation Committee score, along with thigh muscle strength measurements evaluated through isokinetic and isometric testing of flexor and extensor muscle groups.

### Inclusion and exclusion criteria

We included randomized controlled trials (RCTs) and prospective cohort studies involving human subjects who have undergone ACLR, with full-text articles available. There were no restrictions regarding the year of publication.

We excluded studies involving non-operative treatment for ACL rupture, and any systematic reviews, meta-analyses, retrospective cohort studies, case reports, small case series, studies with poor methodological quality, non-English publications, animal studies, and publications on skeletally immature participants.

### Search strategy

A comprehensive and systematic literature search was conducted to identify studies evaluating different rehabilitation protocols following ACLR, aiming to determine the most effective approach. The databases searched include Web of Science, PubMed, Ovid MEDLINE, and Cochrane, from inception until November 2024. Search terms employed a combination of medical subject headings (MeSH) and keywords related to (‘anterior cruciate ligament reconstruction’ OR ‘ACL reconstruction’ OR ACLR) AND (‘home-based’ OR home OR ‘self-directed’) AND (supervised OR ‘clinic-based’ OR ‘in-person’) AND (‘rehabilitation protocol’ OR ‘physical therapy’ OR ‘postoperative rehabilitation’) AND (‘comparative study’ OR ‘effectiveness’ OR ‘outcomes’). Electronic search strategy is provided in (Supplementary Material, Appendix 1 (see section on Supplementary materials given at the end of the article)).

### Study selection

Two independent reviewers screened the initially identified studies based on pre-specified inclusion and exclusion criteria, using Rayyan.AI. Titles and abstracts were evaluated for relevance. Studies meeting the inclusion criteria were thoroughly examined by the review team. A third reviewer resolved any discrepancies.

### Data extraction

Two reviewers independently extracted data using a predesigned Excel datasheet. The extracted data included study characteristics (author, publication year, study design), participant demographics (sample size, age, sex distribution, BMI), details of the rehabilitation protocol (timing, duration, frequency, specific exercises), and outcome measures (subjective knee outcomes, functional knee measures, quadriceps, and hamstring strength measures).

### Assessment of study quality

The risk of bias was assessed using the Cochrane Collaboration risk of bias (RoB) tool (22). Studies were categorized as having low, high, or unclear risk of bias. Following the meta-analyses, the quality of evidence was evaluated using the GRADE (grading of recommendations assessment, development and evaluation) framework. The certainty of the evidence for the outcomes was assessed across five domains (risk of bias, consistency, precision, directness, and publication bias) and rated as ‘high’ or downgraded to ‘moderate,’ ‘low,’ or ‘very low’ quality of evidence (23).

### Qualitative synthesis

To provide a qualitative analysis of the findings and study characteristics, summary tables were generated using data from the relevant studies. The most appropriate method for utilizing the data from the included studies was selected after data retrieval for the systematic review.

### Quantitative synthesis

For meta-analytical data analysis, RevMan (Review Manager) v5.4 (Nordic Cochrane Center, Cochrane Collaboration, Denmark) was used when outcomes were available for pooled analysis in two or more studies. We calculated the standardized mean difference (SMD) with 95% confidence intervals (CIs) for continuous outcomes. When medians and ranges were reported, Hozo’s method was applied to estimate the mean and standard deviation (SD) (24). If numerical data were not explicitly reported in the text, we tried to estimate the data if available in the graphs/figures. If there were no graphs/figures, we tried to contact the authors; if the authors did not respond, the study was excluded from the meta-analysis and reviewed narratively. We did not perform statistical imputation for entirely missing outcome data (e.g. missing group-level results) in order to avoid introducing bias through assumptions. However, when partial data (e.g. missing SDs but available means) were available, standard estimation techniques based on available data were employed in accordance with Cochrane guidelines. Statistical heterogeneity was assessed using the *I^2^* statistic, with heterogeneity categorized as insignificant (0–40%), moderate (30–60%), substantial (50–90%), or considerable (75–100%) (22). If significant heterogeneity was detected, a random-effects model was employed instead of a fixed-effects model.

## Results

### Search strategy

**Figure 1:**
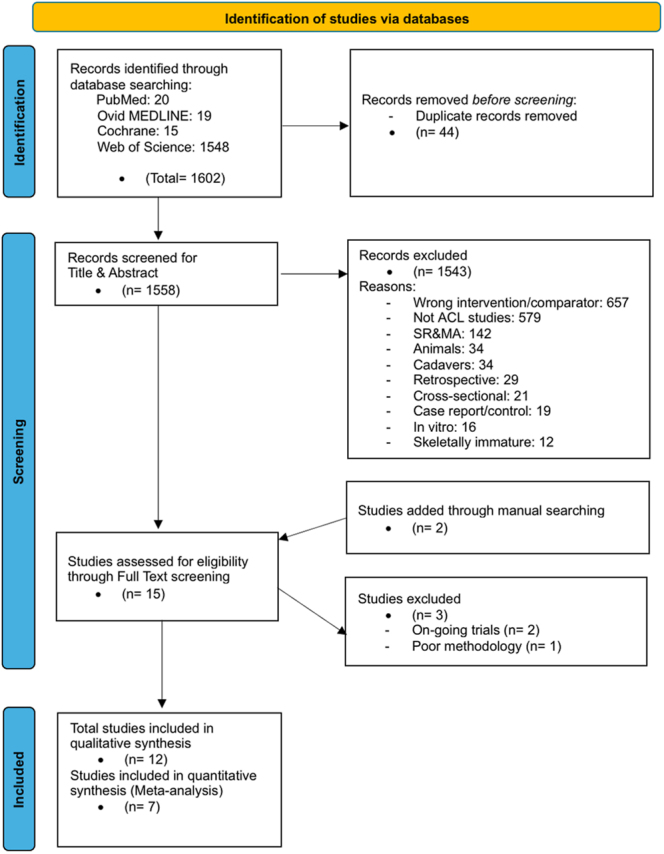
Prisma flow diagram demonstrating the search strategy for the included twelve studies (15, 16, 17, 18, 28, 29, 30, 31, 32, 33, 34, 35).

The flow diagram in Fig. 1 (PRISMA flow diagram) outlines the process of study selection.

A total of 1,602 studies were identified. Following the removal of duplicates (*n* = 44) and the application of inclusion criteria (Table 1), titles and abstracts were screened, excluding 1,587 articles for the following reasons: wrong intervention/comparator (*n* = 657), not ACL studies (*n* = 579), systematic reviews (*n* = 142), animals (*n* = 34), cadavers (*n* = 34), retrospective (*n* = 29), cross-sectional (*n* = 21), case report/control (*n* = 19), *in vitro* (*n* = 16), and skeletally immature (*n* = 12).

**Table 1:** Inclusion and exclusion criteria.

Inclusion criteria	Exclusion criteria
Randomized controlled trials (RCTs) and prospective cohort studies	Systematic reviews, meta-analyses, retrospective cohort studies, case reports, small case series (fewer than ten patients), letters to the editor, or studies with poor methodological quality
Human subjects who have undergone ACL reconstruction	Non-English language publications
Availability of full text	Non-operative treatment for ACL rupture
	Non-human publications
	Publications on skeletally immature participants

After adding two studies through manual searching, we ended up with 15 studies requiring full-text review. Three studies were excluded after initial inclusion: two were ongoing trials where only the study protocols had been published (25, 26), and one was excluded due to poor methodology. The methodological concerns included a lack of detailed description of methods, absence of baseline measurements, no information on the reliability and validity of the tools used to measure outcomes, and insufficient robustness in the statistical analysis, with no justification for the choice of statistical methods (27). This brought the total number of studies included in the qualitative synthesis to 12 (15, 16, 17, 18, 28, 29, 30, 31, 32, 33, 34, 35). Of these, ten were RCTs, and two were prospective cohort studies (16, 35). Seven of the twelve studies were deemed suitable for meta-analysis (16, 17, 18, 28, 32, 33, 34). Two studies did not report the SD for the outcomes of interest (29, 35), and two others did not report the exact outcomes of interest (15, 31).

### Participant characteristics

A summary of the study characteristics is showed in (Table 2). The twelve included studies comprised a total of 711 participants, of which 68.2% (*n* = 485) were males. One study (17) exclusively included male participants. The average age across all studies was 28.3 years. The mean duration of postoperative rehabilitation was 8.5 months in nine studies, with one study (15) reporting an average duration of 38 months, another (18) with 31.1 months, and Schenck *et al.* reporting an average of 21.6 months.

**Table 2:** Study characteristics, graft, follow-ups, and visits.

Study/groups	Year	Design	Subjects, *n*		Characteristics					Visits post-op
			All	Males	Age*	BMI	ACLR graft	FU, mo	IOT, mo	
Schenck *et al.* (29)	1997	RCT					BPTBg	21.6 (12–48)	N/R	
HBR			22	17	23.5	N/R				Average: 3
SVR			15	11	23.5	N/R				3/week for 6 weeks (average: 14.2)
Beard *et al.* (33)	1998	RCT					BPTBg	3, 6	61 (12–132)	
HBR			13	10	27	N/R				1/week initially; then 1/month later
SVR			13	11	29	N/R				2/week for 18 weeks
Fischer *et al.* (35)	1998	PCS					BPTBg + BPTBg	0.25, 1.5, 3, 4.5, 6	1.5–216	
HBR			27	16	32	N/R				6
SVR			27	13	27	N/R				Average: 20
Grant *et al.* (31)	2005	RCT					BPTBg	3	N/R	
HBR			73	47	29.1	27				4 therapy sessions
SVR			72	38	29.5	27				17 therapy sessions
Ugutmen *et al.* (18)	2008	RCT					Hg	31.1 (12–66)	34.3 m	
HBR			52	52	31.5	N/R				N/R
SVR			52	52	31.5	N/R				N/R
Revenäs *et al.* (34)	2009	RCT					BPTBg + Hg	6, 12	15.5 (3–177)	
HBR			27	16	24	N/R				N/R
SVR			24	17	21	N/R				13 visits
Grant *et al.* (15)	2010	RCT					BPTBg	38	26–52	
HBR			40	27	30.8	27.3				N/R
SVR			48	23	30.3	26.2				N/R
Hohmann *et al.* (28)	2011	RCT					N/A	3, 6, 9, 12	N/R	
HBR			20	14	27	25.5				1 at 3, 6.9, 12 months
SVR			20	16	28	24.5				1/week for 6 weeks; biweekly until 6 months; 1/month for 9 months
Przybylak *et al.* (30)	2018	RCT					BPTBg + Hg	12 m	N/R	
HBR			25	19	27	24.8				5
SVR			25	18	34	25.5				47
Lim *et al.* (32)	2019	RCT					Hg	6 m	N/R	
HBR			15	9	38.8	24.1				N/R
SVR			15	10	32.2	25.5				N/R
Rhim *et al.* (16)	2020	PCS					Hg	6, 12	N/R	
HBR			13	9	28.6	25.4				4 at weeks 2 & 6 and at months 3 & 6
SVR			13	10	27.1	27.5				1/week for 3 months; bi-weekly after 3 months
Syed *et al.* (17)	2024	Pilot					Hg	8 m	N/R	
HBR			30	15	24.9	24.7				5–12
SVR			30	15	22.4	23.2				40–64

RCT, randomized control trials; HBR, home-based rehabilitation; SVR, supervised rehabilitation; BMI, body mass index; mo, months; ACLR, anterior cruciate ligament reconstruction; BPTBg, bone-patellar tendon bone graft; Hg, hamstring graft; N/R, not reported; PCS, prospective cohort study; FU, follow-up; IOT, injury-to-operation time; post-op, post-operation.

* Mean age in years.

Five of the 12 studies involved patients who received a bone-patellar tendon-bone (BPTB) graft (15, 29, 31, 33, 35), while four studies included patients with hamstring grafts (16, 17, 18, 32). Two studies involved a mixed cohort of patients with either BPTB or hamstring grafts. Hohmann *et al.* did not specify the type of graft used for ACL reconstruction (30, 34).

All studies compared HBR to supervised rehabilitation (SVR). The average number of participants per group was approximately 30, with group sizes ranging from 13 to 73. The average body mass index (BMI) was reported in seven of the twelve studies; five studies did not report BMI (17, 25, 29, 30, 31). The average BMI in the HBR group was 25.5, while the SVR group had an average BMI of 25.7. The rehabilitation protocols for each study are provided in (Supplementary Material, Appendix 2).

### Risk of bias

**Figure 2:**
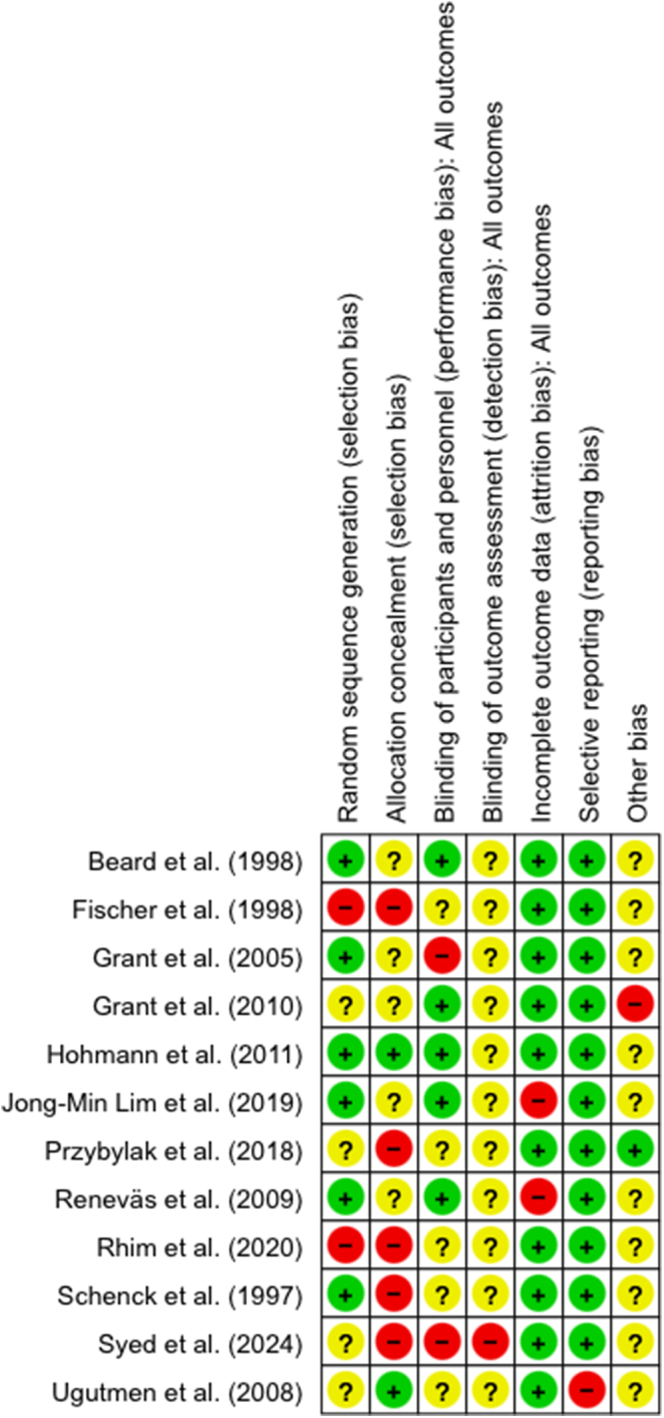
Risk of bias summary for the included studies (15, 16, 17, 18, 28, 29, 30, 31, 32, 33, 34, 35).

The Cochrane Collaboration’s risk of bias tool (RoB) was employed to assess the risk of bias in the included studies, classifying each domain as either low risk (green ‘+’), unclear/some risk (yellow ‘?’), or high risk (red ‘−‘). A summary of the risk of bias across the twelve studies is depicted in Fig. 2. Five studies showed high risk in 'Selection bias' (16, 17, 30, 35, 36), with two (16, 17) showing high risk in both 'Random Sequence Generation' and 'Allocation Concealment'. Only Hohmann *et al.* (28) had low risk in both. For 'Performance bias', two studies (17, 31) had high risk, while five (16, 18, 29, 30, 35) were unclear. The remaining five studies exhibited low risk. 'Detection bias' was generally unclear, except Syed *et al.* (17), which had high risk. 'Attrition bias' was high in two studies (32, 34) and low in the rest. All studies except Ugutmen *et al.* (18) were low risk for Reporting bias. In 'Other bias', most studies lacked protocol access, except Pryzybylak *et al.* (30), and Grant *et al.* (15) had a potential conflict of interest (15).

Overall, ten studies showed moderate to high bias risk, with only Beard *et al.* (33) and Hohmann *et al.* (28) showing low to moderate risk.

### Outcome measures

All twelve included studies compared HBR protocol to SVR protocol. They evaluated subjective outcome scores, such as the Lysholm score (16, 18, 28, 33, 34, 37), TAS (17, 28, 30, 33), and International Knee Documentation Committee (IKDC) score (15, 17, 18, 33, 34), as well as strength measures for both quadriceps and hamstrings using isometric (17, 28) and isokinetic tests (16, 28, 32, 33).

Regarding the IKDC score, Syed *et al.* (17) was the only study to report a significant difference, favoring the SVR group at 8 months (81.8 vs 68.4, *P* = 0.002). The other four studies showed no significant differences between HBR and SVR groups, with *P*-values indicating non-significance (*P* = 0.8, *P* = 0.4, and *P* = 0.76).

***Lysholm Score*** is a 100-point system designed to assess knee function across eight domains: pain, swelling, instability (‘giving way’), locking, limping, stair climbing, squatting, and the need for support. Scores of 95–100 are classified as excellent, 84–94 as good, 65–83 as fair, and below 65 as poor (37). It was reported in six studies, all showing significant improvement from baseline to final evaluation. Four studies found no significant differences between home-based and supervised rehabilitation groups, while Rhim *et al.* and Reneväs *et al.* (16, 34) reported significant group differences.

Reneväs *et al.* observed a 19% improvement in HBR and only 1% in SVR at 12 months, favoring HBR (*P* = 0.008). In contrast, Rhim *et al.* showed higher scores in the SVR group at 12 months (*P* < 0.001). Other studies, including Ugutmen *et al.* and Beard *et al.* found no significant differences between groups at final evaluation.

**Figure 3:**
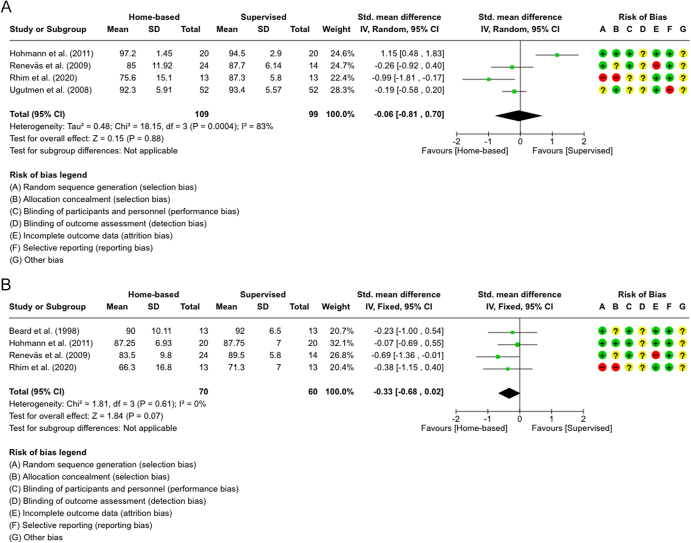
(A) Forest plot showing Lysholm scores at 6 months follow-up in four studies (15, 17, 24, 30). (B) Forest plot showing Lysholm scores at 12 months follow-up in four studies (15, 24, 29, 30).

Figure 3 presents forest plots for the Lysholm score at 6 and 12 months or more after ACLR, as assessed in five studies (16, 18, 28, 33, 34). Meta-analysis at 6 months (Fig. 3A) included four studies (16, 18, 28, 34), mostly favoring SVR, but the effect was statistically insignificant (SMD = −0.06, *P* = 0.88). A random-effects model was applied due to high heterogeneity (*I^2^* = 83%), resulting in low evidence quality (Table 2). The 12-month analysis (Fig. 3B), which was done in four studies as well (16, 28, 33, 34), favored SVR. A fixed-effects model was used due to the absence of heterogeneity (*I^2^* = 0%). Although the effect was marginally non-significant (SMD = −0.33, *P* = 0.07), it was rated as moderate-quality evidence (Table 3).

**Table 3:** The table summarizes the GRADE assessments, highlighting the confidence in the estimates of effect.

Outcomes	Time, months	Quality assessment					Summary of findings				
							Participants, *n*		Effect		Certainty in estimates
		ROB*	Consistency^†^	Precision^‡^	Directness^§^	Pub. bias	Home-based	Supervised	SMD (95%CI)	Result	
Lysholm score	6	NSL	SL	SL	NSL	Undetected	109	99	−0.06 (−0.81 to 0.70)	FSG	Low
Lysholm score	12 & more	NSL	NSL	SL	NSL	Undetected	70	60	−0.33 (−0.68 to 0.02)	FSG	Moderate
Isometric quadriceps strength	8–9	SL	NSL	SL	NSL	Undetected	50	50	−0.34 (−0.74 to 0.06)	FSG	Low
Isometric hamstring strength	8–9	SL	NSL	NSL	NSL	Undetected	50	50	−0.48 (−0.88 to −0.08)	FSG	Moderate
Isokinetic quadriceps strength	6	SL	SL	SL	NSL	Undetected	48	48	−0.61 (−2.10 to 0.88)	FSG	Very low
Isokinetic hamstring strength	6	SL	NSL	NSL	NSL	Undetected	48	48	0.00 (−0.40 to 0.40)	Neutral	Moderate
Isokinetic quadriceps strength	12	SL	NSL	SL	NSL	Undetected	33	33	−0.39 (−0.88 to 0.10)	FSG	Low
Isokinetic hamstring strength	12	SL	NSL	NSL	NSL	Undetected	33	33	−1.34 (−1.89 to −0.79)	FSG	Moderate

CI, confidence interval; FSG, favors supervised group; SL, serious limitation; NSL, no serious limitation; SMD, standardized mean difference; Rob, risk of bias; Pub. Bias, publication bias.

* Risk of bias was downgraded if the high-risk domain was likely to affect the outcome.

^†^ Consistency was downgraded if the heterogeneity was statistically significant [*P* < 0.05].

^‡^ Precision was downgraded if the confidence interval crossed zero, indicating no difference.

^§^ Directness was downgraded if different measures were used.

### Tegner Activity Scale (TAS)

Four studies in the review reported the TAS, which is a commonly used tool to assess an individual’s level of physical activity. It ranges from 0 (disability due to knee problems) to 10 (competitive sports at the international level) (38).

In Przybylak *et al.* (30), the SVR group improved from a TAS score of 5 pre-surgery to 6 at final follow-up, while the HBR group remained at 5. This improvement in the SVR group was significant (*P* < 0.001), with a notable difference between groups at 12 months (*P* = 0.003).

Syed *et al.* (17) reported that both groups experienced a slight drop in TAS scores post-surgery, with the SVR group going from 8 to 7 and the HBR group from 8 to 6 at 8 months, though scores remained slightly higher in the SVR group.

Beard *et al.* (33) used a modified TAS score (activity level as a percentage of pre-injury). The HBR group scored 66% (SEM = 4.5), and the SVR group 72% (SEM = 4.5), with no significant difference (*P* = 0.41).

Hohmann *et al.* (28) reported the HBR group’s TAS score increased from 4 (range 2–8) pre-surgery to 5 (range 3–10) at 12 months, while the SVR group increased from 3 (range 2–8) to 6 (range 3–8). There was no significant difference between groups at 12 months.

### Isometric extensor (quadriceps) and flexor (hamstring) symmetry indexes

Regarding the isometric symmetry index derived from quadriceps strength, Hohmann *et al.* reported a significant difference favoring the HBR group for quadriceps symmetry at 3 months, with a mean of 71.1 in the HBR group and 56.4 in the supervised rehabilitation group (*P* = 0.01). However, this difference was not significant at later follow-ups. For hamstring symmetry, there were no significant differences between the two groups, with fluctuating results (*P* = 0.2).

For isometric quadriceps and hamstring strength, Hohmann *et al.* reported higher quadriceps peak torque in the HBR group compared to the SVR group, but the difference was not significant. Syed *et al.* found the SVR group had slightly higher quadriceps strength than the HBR group, but again, no significant difference was observed. Similarly, for hamstring strength, both groups showed continuous improvement, but no significant differences were found at the final assessment.

**Figure 4:**
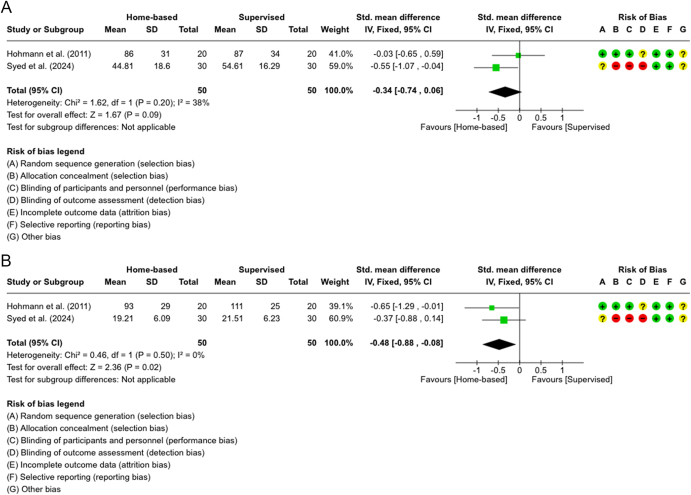
(A) Forest plot showing isometric strength measures at 8–9 months for quadriceps (extensor) muscles in two studies (16, 24). (B) Forest plot showing isometric strength measures at 8–9 months for hamstring (flexor) muscles in two studies (16, 24).

The meta-analysis in Fig. 4A pooled data from these studies, revealing that for quadriceps strength, both groups showed similar results, with a slight trend favoring the supervised group (SMD = −0.34, *P* = 0.09). However, the analysis was statistically insignificant. A fixed-effect model was applied because of the low heterogeneity (*I*^2^ = 38%). For hamstring strength in Fig. 4B, the supervised group showed a significant advantage (SMD = −0.48, *P* = 0.02), with no heterogeneity between studies; therefore, the fixed-effect model was used. The quality of evidence for quadriceps strength was rated as low due to risk of bias and imprecision, while the evidence for hamstring strength was rated as moderate due to the same concerns (Table 3).

### Isokinetic concentric extensor and flexor symmetry indexes

Hohmann *et al.* observed that the isokinetic quadriceps symmetry index significantly improved over time in the SVR group, from 63.7 at baseline to 82.7 at 12 months (*P* = 0.04). In contrast, the HBR group showed a non-significant increase, from 75.5 at baseline to 79.5 at 12 months (*P* = 0.15). Both groups showed non-significant improvements in the isokinetic hamstring symmetry index between 3 and 12 months.

### Isokinetic concentric extensor and flexor strengths

These were reported in four studies (16, 28, 32, 33). They found no significant differences in quadriceps peak torque between the two groups at 12 months, with *P*-values ranging from 0.1 to 0.5. In contrast to quadriceps strength, hamstring peak torque showed significant differences favoring the SVR group by Hohmann *et al.* and Rhim *et al.* (*P* < 0.05). However, JM Lim *et al.* and Beard *et al.* found no significant differences for hamstring strength.

**Figure 5:**
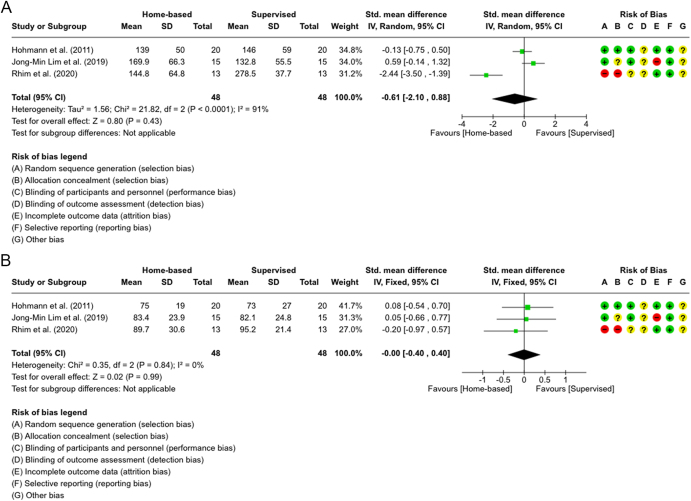
(A) Forest plot showing isokinetic strength measures at 6 months for quadriceps (extensor) muscles in three studies (15, 24, 28). (B) Forest plot showing isokinetic strength measures at 6 months for hamstring (flexor) muscles in three studies (15, 24, 28).

**Figure 6:**
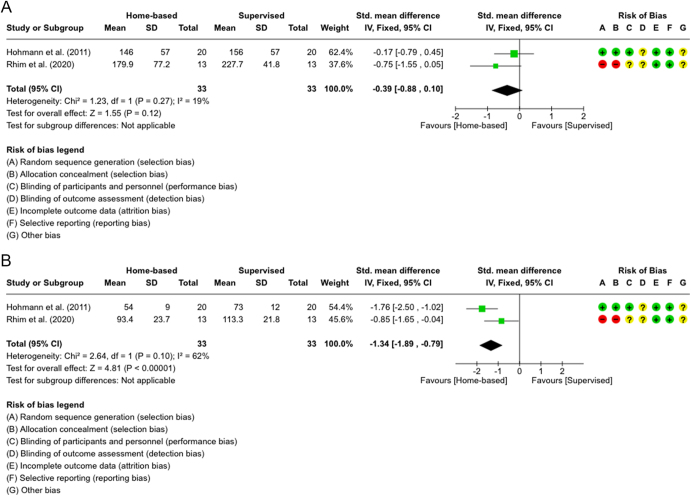
(A) Forest plot showing isokinetic strength measures at 12 months for quadriceps (extensor) muscles in two studies (15, 24). (B) Forest plot showing isokinetic strength measures at 12 months for hamstring (flexor) muscles in two studies (15, 24).

The meta-analysis (Figs 5 and 6) examined isokinetic strength at 6 months (16, 28, 32) and 12 months (16, 28). For extensor strength at 6 months (Fig. 5A), only JM Lim *et al.* showed a significant result favoring the SVR group, but the overall pooled analysis was not statistically significant (SMD = −0.61; 95% CI: −2.10 to 0.88, *P* = 0.43). We used a random-effect model since the heterogeneity was significantly high (*I^2^* = 91%). The evidence quality was rated very low because of bias and inconsistency (Table 3). For flexor strength at 6 months (Fig. 5B), the overall effect was statistically insignificant (SMD = 0.00; 95% CI: −0.40 to 0.40, *P* = 0.99). A fixed-effect model was used because of the low heterogeneity (*I^2^* = 0%). The quality of evidence was rated moderate due to risk of bias (Table 3).

For extensor strength at 12 months (Fig. 6A), both studies favored the SVR group, though the pooled analysis remained slightly insignificant (SMD = −0.39; 95% CI: −0.88 to 0.10, *P* = 0.12). A fixed-effect model was applied because of the low heterogeneity (*I^2^* = 19%). The quality of evidence was low due to bias and imprecision (Table 3). For flexor strength (Fig. 6B), the result was significant (SMD = −1.34; 95% CI: −1.89 to −0.79, *P* < 0.00001), strongly favoring the SVR group. A fixed-effect model was used once again because the heterogeneity was insignificant (*I^2^* = 62%). The quality of evidence was moderate due to risk of bias (Table 3).

It is also noteworthy that quadriceps ratio (15, 31), hamstring ratio (15, 17, 31), squat analysis, and hip adductors and abductors (17) showed significant improvements over time in both groups, but no significant differences were reported between HBR and SVR.

### Other outcomes

Knee Injury and Osteoarthritis Outcome Score (KOOS): Przybylak *et al.* found significant KOOS improvements in both groups. The SVR group’s score increased from 60 at baseline to 100 at the last visit (*P* < 0.001), while the HBR group improved from 70 to 85 (*P* < 0.008). At 12 months, the SVR group had significantly better KOOS scores than the HBR group (*P* < 0.001). Quality of life (QoL): Przybylak *et al.* also reported QoL improvements: the SVR group’s score rose from 44 to 94 (*P* < 0.001), while the HBR group increased from 44 to 75 (*P <* 0.001), with a significant difference favoring the SVR group at 12 months (*P* < 0.001). Contrarily, Grant *et al.* (15) observed higher QoL in the HBR group (80 vs 69.9 for SVR, *P* < 0.02) at the last visit (15).

Proprioception: Lim *et al.* found a significant improvement in proprioception in the SVR group, whereas the HBR group’s improvement was not significant (*P* > 0.05) (32). The overall postoperative proprioceptive outcomes favored the SVR group (*P* < 0.05). Return to sports: Syed *et al.* reported that a higher percentage of the SVR group returned to their pre-injury level of sports (76.6%) compared to the HBR group (53.3%), with significant differences between the groups (*P* = 0.036). In addition, 16.6% of SVR and 30% of HBR participants returned to lower levels, while 6.6% of SVR and 16.6% of HBR participants did not return to sports. Knee laxity (KT-1000): reported in five studies, knee laxity measurements showed no significant differences between the SVR and HBR groups (e.g., *P* = 0.09, *P* = 0.22, *P* = 0.1 across studies).

Other functional measures, such as Sickness Impact Profile and quadriceps atrophy (Schenck *et al.*): No significant differences between groups. Single hop, timed hop, and vertical jump: Significant improvements were noted at the last evaluation in each study, though no statistically significant differences were found between HBR and SVR groups. Range of motion: one study (31) showed significant differences favoring the HBR group in knee flexion (*P* < 0.03) and extension (*P* < 0.02).

## Discussion

Twelve studies with 711 participants assessed outcomes such as the Lysholm score, TAS, and strength metrics, with seven studies included in the meta-analysis. Six studies examined the Lysholm score (16, 18, 28, 29, 33, 34). Most found no significant group differences, except Rhim *et al.* and Reneväs *et al.* Reneväs *et al.* reported a higher Lysholm score for the home-based group at 12 months, likely due to a lower baseline score and a high noncompliance rate in the supervised group. Rhim *et al.* found the opposite, with better scores in the supervised group.

The meta-analysis for the Lysholm score provided low-to-moderate quality evidence at 6 months (16, 18, 28, 34) and 12 months (16, 28, 33, 34) (Table 3), with results favoring the supervised group slightly, though statistically insignificant (Fig. 3). Four studies evaluated the TAS (17, 29, 30, 33), with most showing no significant group differences. However, Przybylak *et al.* found notable improvement in the supervised group at 12 months, likely due to better compliance. Differences in rehabilitation duration – 12 months in Przybylak *et al.*, 6 months in Beard *et al.*, 8 months in Syed *et al.*, and 12 months again in Hohmann *et al.* – and participant characteristics across studies may have influenced outcome discrepancies.

For isometric strength and symmetry indexes of the quadriceps and hamstrings, two studies were reviewed (17, 28). Hohmann *et al.* found a significant difference favoring the home-based group for quadriceps symmetry at 3 months, but all other follow-ups (whether symmetry indexes or strength measures) showed no significant group differences. One of the possible explanations is that the home-based group might feel more responsible for not having direct guidance, motivating them to be more engaged in their rehabilitation. In contrast, the supervised group might rely more on supervision and feel less accountable, slowing down their progress. Quadriceps strength analysis slightly favored the supervised group (not statistically significant), while hamstring strength analysis significantly favored the supervised group (Fig. 4), with no heterogeneity but low quality of evidence due to bias and imprecision. This may be attributed to the higher intensity and regularity of supervised exercises, as supported by Hohmann *et al.* Such activities and exercises include step-ups, increased cycling resistance, and sport-specific drills, all of which require active quadriceps engagement. In addition, supervised settings likely promote greater exercise adherence, proper technique, and regular progression, which are critical for rebuilding quadriceps strength post-ACLR.

Isokinetic strength was reported in four studies (16, 28, 32, 33), and most showed no group differences, except Hohmann *et al.* and Rhim *et al.*, which found significant improvements in supervised hamstring strength at 12 months. This can be due to the lack of specialized equipment and optimal techniques for strengthening the hamstring muscles at home, whereas machines and professional guidance are most likely available for the supervised group. Meta-analyses for three studies at 6 months (Fig. 5) and two studies at 12 months (Fig. 6) showed that the quality of evidence was rated as very low-to-moderate for the 6-month analysis and low-to-moderate for the 12-month analysis (Table 3). While the quadriceps pooled analysis in Fig. 5 favored the supervised group, it was statistically insignificant. The hamstring analysis effect was neutral, showing no preference between the groups. In contrast, both plots in Fig. 6 suggested that the supervised group was superior, with the hamstring pooled analysis showing a strong statistically significant overall effect, with moderate heterogeneity but still insignificant.

Interestingly, an overview of systematic reviews was conducted by Culvenor *et al.* (39) evaluating the effectiveness of rehabilitation interventions following ACL and/or meniscus tear. The review suggested that there is a low level of evidence in improving symptomatic and functional outcomes after ACL rehabilitation interventions. The highest level of evidence (moderate certainty) was found for the effectiveness of bracing, neuromuscular electrical stimulation, and open and/or closed kinetic chain exercises. The review also reported that HBR was as effective as supervised rehabilitation for improving quadriceps and subjective outcomes, which supports several studies that suggested supervised rehabilitation may not always outperform home-based programs across orthopedic conditions (21, 40, 41, 42), which ultimately supports our findings for this study. However, longer supervised programs (over 6 months) seem more effective in helping patients meet return-to-sports criteria (21, 43, 44). Gamble *et al.* (2020) (19) conducted a systematic review and meta-analysis comparing intensive supervision and less supervised rehabilitation after ACLR and found no significant differences between both protocols for athletes, based on low to very low certainty of evidence. Those findings align well with our results, where no significant differences between home-based and supervised protocols can be observed, though supervised rehabilitation may be preferable for maximizing strength gains and achieving preinjury levels. In line with Uchino *et al.*, our findings support maintaining some professional oversight to ensure correct technique and progression.

## Limitations

A major limitation of this review is the heterogeneity in outcome measures across studies. The presence of moderate heterogeneity in some pooled analyses, particularly in the 12-month hamstring strength outcome, limits the certainty with which the findings can be generalized. This variability may reflect differences in study protocols, rehabilitation intensity, patient adherence, or assessment methods across the included studies. As a result, the observed effects – especially those favoring supervised rehabilitation – should be interpreted with caution. In addition, variations in study quality, with some showing high risk of bias, may affect the reliability of the findings, potentially skewing effectiveness estimates. The absence of long-term outcomes also limits insights into the lasting benefits of both rehabilitation protocols.

## Conclusion

This review indicates that HBR is generally as effective as supervised protocols for subjective knee function. However, supervised rehabilitation shows a slight advantage in strength gains, which may be beneficial for athletes or individuals with high physical demands. While both approaches are viable, future research should prioritize high-quality, long-term trials targeting specific populations, such as athletes, and comparing them to the general population.

## Supplementary materials



## ICMJE Statement of Interest

No benefits in any form have been received or will be received from a commercial party related directly or indirectly to the subject of this article.

## Funding Statement

The authors did not receive funding from a third party, institution, organization, or company.

## Author contribution statement

Both WDK and KMA contributed equally to the conception and design of the study, performed database searches, independently screened studies for inclusion, and conducted data extraction and analysis. Both authors participated in the interpretation of results, drafting of the manuscript, and critical revisions for intellectual content. Each author has approved the final version of the manuscript and agrees to be accountable for all aspects of the work.
