# Supplementary material for: Comparison between home-based and supervised rehabilitation protocols after anterior cruciate ligament reconstruction: a systematic review and meta-analysis
Source: EFORT Open Rev. 2025 Sep 4;10(9):695–708. doi: 10.1530/EOR-2024-0216 (PMC12412367; doi:10.1530/EOR-2024-0216)
Supplement: Supplementary file 1 [file supplementary_materials.pdf]

Supplementary Material

Web of Science

Appendix 1. Search Strategy

(((((((((AB=(Anterior cruciate ligament reconstruction)) OR AB=(ACL reconstruction)) OR AB=(ACLR)) AND AB=(Home based)) OR AB=(Home)) AND AB=(Supervised)) OR AB=(Clinic based)) AND AB=(Rehabilitation protocol)) OR AB=(Physical therapy)) AND AB=(Comparison)

-[Exact search] option= 2767

-[Open access] filter= 1548

PubMed

("ACL Reconstruction" OR "Anterior Cruciate Ligament Reconstruction" OR ACLR) AND ("Home Based" OR Home OR "Self Directed") AND (Supervised OR "Clinic Based" OR "In Person") AND ("Rehabilitation protocol" OR "Physical therapy" OR "Postoperative rehabilitation") AND ("Comparative Study" OR "Effectiveness" OR "Outcomes" OR "Comparison")

Returned 20 articles.

Cochrane library

("Anterior Cruciate Ligament Reconstruction" OR " ACL Reconstruction" OR ACLR) AND ("Home-Based" OR Home OR "Self-Directed") AND (Supervised OR "Clinic-Based" OR "In-Person") AND ("Rehabilitation protocol" OR "Physical therapy" OR "Postoperative rehabilitation") AND ("Comparative Study" OR "Effectiveness" OR "Outcomes").

Returned 15 articles.

Appendix 2. Rehabilitation Protocols

Table of Rehabilitation Protocols Details		
	Rehabilitation protocol	
	Home-based	Supervised
**Schenck et al. (1997)[1]**	1. Exercise Types: Exercises can include walking, stair climbing, and functional tasks that can be performed at home.<br>2. Frequency and Duration: The protocol generally involves a lower Frequency and Duration compared to supervised group exercises.<br>3. Intensity: Exercises are typically of moderate Intensity, focusing on maintaining activity levels rather than high-Intensity training.<br>4. Supervision: Patients perform Exercises independently at home, with periodic check-ins with healthcare providers.	1. Exercise Types: The exercises are more structured and varied, including resistance training, aerobic exercises, and specific therapeutic exercises guided by professionals.<br>2. Frequency and Duration: This protocol involves more frequent and longer sessions compared to home-based exercises.<br>3. Intensity: Higher intensity with progressive difficulty to improve strength, endurance, and functional abilities.<br>4. Supervision: Constant supervision and guidance from healthcare professionals during exercise sessions to ensure correct form and adjust intensity as needed.
**Beard et al. (1998)[2]**	Patients in this group perform all their exercises at home or using alternative commercial/private facilities.<br>- They attend physical therapy sessions only for assessments, education, modifications, and progression of their treatment plan.<br>- The frequency of follow-up appointments is determined by the treating therapist, based on clinical discretion. Generally, more frequent visits (once per week) are recommended early in the rehabilitation process, with less frequent visits (up to once per month) as rehabilitation progresses.	In addition to the home-based exercises identical to those in Group H, patients in this group attend a supervised physical therapy knee class twice a week.<br>- These supervised sessions include "proprioceptive" or functional exercises, which are designed to enhance the rehabilitation process. Some exercises may also be updated from other sources.<br>- Like Group H, the content and progression of exercises are managed by a therapist, and attendance at these supervised sessions begins between 4 to 6 weeks post-operation.

**Fischer et al. (1998)[3]**	This group was prescribed 6 supervised physical therapy visits in the first 6 months after surgery.<br>- They were given home exercise booklets containing descriptions, diagrams, and timing of specific exercises that could be used in the home.<br>- The rehabilitation program for the home-based group was divided into 4 phases: (1) restoration of ROM, (2) beginning functional strengthening, (3) advanced functional strengthening, and (4) improvement of speed and agility.	This group included 24 physical therapy appointments in the first 6 months (Fig 1).<br>- It was anticipated that the actual course of physical therapy would vary from patient to patient, so the controlled variable was the number of prescribed visits, not the number of actual visits.<br>- All patients in the supervised clinic group were observed for at least 6 months after surgery with clinic visits scheduled at 1, 6, 12, 18, and 24 weeks.<br>- At each visit, patients underwent a standard physical examination of the involved and contralateral knees.
**Grant e al. (2005)[4]**	This group attended an average of 3 to 6 physical therapy sessions.<br>- The program emphasized preoperative education and providing patients with detailed exercise instruction booklets.<br>- Patients were required to follow a structured rehabilitation plan that included:<br>- Early weightbearing.<br>- Knee joint motion exercises.<br>- Low-level graft stress activities.<br>- Progressive strengthening exercises.<br>- Periodic follow-up was necessary to ensure no complications arose and to adjust the rehabilitation plan if needed.	This group attended 14 to 24 physical therapy sessions on average.<br>- This program also included preoperative education and was structured similarly to the home-based program but with more frequent supervised sessions.<br>- The supervised sessions involved:<br>- Close monitoring by physical therapists.<br>- Adjustments to the exercises based on patient progress.<br>- Ensuring correct execution of exercises to prevent injuries and complications.

**Ugutmen et al. (2008)[5]**	1. Phases and Goals:<br>- Phase 1: Pre-operative exercises to achieve full range of motion, reduce edema, and increase extensor muscle strength.<br>- Phase 2: (0-3 weeks post-op) Gain full extension and 90° flexion through closed-chain exercises.<br>- Phase 3: (4-6 weeks post-op) Increase range of motion and return to normal daily activities.<br>- Phase 4: (6 weeks - 4 months post-op) Begin sporting activities.<br>- Phase 5: (4-6 months post-op) Return to sports.<br>- Phase 6: (6-8 months post-op) Return to contact sports.<br><br>2. Implementation:<br>Patients were provided with a home exercise booklet that included descriptions and diagrams of the exercises. They were seen by physical therapists and an orthopedic surgeon weekly for the first six weeks, bi-weekly for the next six weeks, and then monthly for physical examination and measurements.	The supervised group followed the same exercise phases and goals as the home-based group. The major difference was the setting, where the exercises and rehabilitation were conducted under the direct supervision of healthcare professionals at a clinic. This setup allowed for more frequent monitoring and immediate adjustments to the exercise regimen as needed.

**Revenäs et al. (2009)[6]**	* Pre-Operative Phase: Included CKC (e.g., squats, lunges) and OKC (e.g., leg extensions, leg curls) exercises, done at least twice a week, both at home and in a gym.<br>*Post-Operative Phase:<br>- 0-6 weeks: Focused on mobility, muscle activation, and gradual strengthening through exercises like heel slides, isometric quadriceps contractions, and partial squats. Patients had weekly check-ins with a physiotherapist for guidance.<br>- 6 weeks - 6 months: Continued with a self-directed exercise program, incorporating more advanced strength training, proprioceptive exercises, and sport-specific drills, with occasional physiotherapist appointments for adjustments.	* Pre-Operative Phase: Similar to the GT group with CKC and OKC exercises.<br>* Post-Operative Phase:<br>- 0-6 weeks: Similar initial focus as the GT group, under physiotherapist supervision.<br>- 6 weeks - 6 months: Patients attended supervised knee classes, where they performed advanced strength training, proprioceptive exercises, and sport-specific drills in a group setting, under direct physiotherapist supervision.

**Grant et al. (2010)[7]**	1. Exercises: Participants in the home-based group followed a regimen of specific exercises designed to be performed at home. These exercises focused on improving strength, flexibility, and endurance.<br>2. Frequency and Duration: The exercise program was structured to be done several times a week, with each session lasting a set duration to ensure adequate training volume.<br>3. Monitoring and Feedback: Regular check-ins were conducted via phone or video calls to monitor progress and provide feedback. Participants were also encouraged to keep logs of their activities to track their adherence and progress.<br>4. Materials: Participants were provided with instructional materials, including manuals and videos, to guide them through the exercises and ensure proper form and technique.	1. Exercise Sessions: The supervised group followed the same exercises as the home-based group but through scheduled sessions at a rehabilitation center or clinic. These sessions were led by trained professionals who provided real-time guidance and supervision.<br>2. Frequency and Duration: Similar to the home-based group, the supervised sessions were conducted several times a week, with each session having a predetermined duration.<br>3. Personalized Adjustments: The exercises were tailored to each individual's needs and adjusted based on their progress and any difficulties encountered.<br>4. Direct Feedback: Participants received immediate feedback and correction on their exercise technique and performance, ensuring they were performing the exercises correctly and safely.
**Hohmann et al. (2011)[8]**	1. Postoperative Care:<br>- Cold compression with a Cryo/Cuff was used intermittently for 24 hours.<br>- Patients were placed in a ROM brace for 6 weeks and were given crutches.<br>- Immediate postoperative weight-bearing was allowed as tolerated.<br><br>2. Stages and Goals:<br>- Stage I (0–2 weeks): Focus on knee extension, muscle tightening, and bending the knee to 90°.<br>- Stage II (2–6 weeks): Full weight-bearing, gentle squats, exercise bike (50 Watts), calf raises, and hamstring curls.<br>- Stage III (6–12 weeks): Increase knee bend to 120°, increase bike resistance (100 Watts), step-ups, balance work, and pool exercises.<br>- Stage IV (3–6 months): Full knee movement, jogging, sprints, and non-contact sport-specific	1. Postoperative Care:<br>- Same as the home-based group.<br><br>2. Stages and Goals:<br>- The same staged rehabilitation protocol with goals and instructions was followed as in the home-based group.<br><br>3. Implementation:<br>- Patients attended supervised sessions at the Physiotherapy Department. They were seen weekly for the first six weeks, biweekly until six months, and monthly until nine months post-surgery. The same physiotherapist monitored all patients throughout the study.

	skills.<br>- Stage V (from 6 months): Return to sports with full range of movement and good quadriceps strength, with no contact sports until 9 months post-surgery.<br><br>3. Implementation:<br>- Patients received a detailed handout with instructions divided into goals for each stage. They were advised to progress to the next stage only after meeting the previous stage's goals.	
**Przybylak et al. (2018)[9]**	*Phases:<br>1. 0-2 Weeks: Focus on restoring range of motion (initially extension), controlling swelling and pain, preventing post-surgical adhesions, maintaining neuromuscular control, teaching proper movement techniques, and learning correct weight-bearing practices.<br>2. 2-6 Weeks: Work on regaining normal range of motion (full extension and flexion up to 90 degrees), correcting gait patterns, rebuilding muscle strength, and resuming daily activities.<br>3. 6-12 Weeks:Aim for full knee range of motion, return to work and normal daily activities, and introduce exercises for eccentric strength, proprioception, central stabilization, and neuromuscular control.<br>4. 12 Weeks to 12 Months: Focus on returning to recreational sports and preventing issues with the patellofemoral joint.<br><br>*Appointments: This group only met the physiotherapist at the beginning of every phase.	Same exercise choices but have more appointments with a physiotherapist with each one lasting approximately 1.5 hours,

**Jong-Min Lim et al. (2019)**[10]	*Phases:<br>1- Preconditioning (2 weeks): Quadriceps setting, hamstring stretching, full knee extension.<br>2- (2-4 weeks): Quadriceps setting, mini wall squats, straight leg raises, knee flexion up to 90°.<br>3- (4-8 weeks): Stationary cycling, squats, knee flexion/extension with bands, balance exercises.<br>4- (8-12 weeks): Continued cycling, deeper squats, lunges, balance exercises with a board.<br>5- (12-24 weeks): Squats to 90°, full knee flexion/extension, jogging on a treadmill.	The supervised rehabilitation program follows the same phases and exercises as the home-based program.<br>- The main difference is that the exercises are performed under the supervision of a physical therapist, twice per week, with no home exercise program.<br>- This group received full education with pictures on the first visit and conducted exercises in individual sessions under a physical therapist's supervision.

**Rhim et al. (2020)**[11]	Patients are provided with instructional videos that demonstrate the rehabilitation process and exercises.<br>- The rehabilitation is divided into five stages, with each stage being introduced during hospitalization and follow-up visits at 2 weeks, 6 weeks, 3 months, and 6 months.<br>- Patients progress to the next stage only if they meet the goals of the previous stage, which are checked by physical therapists during follow-up visits.	Patients visit the sports medicine center once a week for 3 months for supervised rehabilitation.<br>- A physical therapist follows up with the patients throughout the study duration.<br>- After the initial 3 months, follow-ups occur bi-weekly or at longer intervals, depending on the therapist's discretion.<br>- The supervised rehabilitation follows the same stages as the home-based program, but with the added benefit of regular supervision and adjustments by a therapist.

**Syed et al. (2024)[12]**	*Phases:<br>1- (0-2 Weeks): No brace, partial weight-bearing, pain management, ROM: 90° flexion to 0° extension.<br>2- (Week 2–6): Full weight-bearing, full ROM, continued pain management.<br>3- (Week 6–12): Proprioception and agility training, swimming, cycling, gymnastics, functional performance, joint stability.<br>4- (Month 3–6): Sport-specific, non-competitive training, strength, power, endurance, agility.<br>5- (Month 6–9): Sport-specific rehab, team training, confidence, motivation, ongoing injury prevention, and Return to Sport (RTS).<br><br>*Sessions and Monitoring:<br>- Unsupervised home exercises with written instructions and pictorial representations.<br>- Minimum of two exercise sessions per week.<br>- Periodic assessments and regular communication (phone/video calls) for adherence monitoring.	Same exercise choices as the other group.<br><br>*Sessions and Monitoring:<br>- Supervised physical therapy classes twice a week in an outpatient rehabilitation clinic.<br>- Each session lasts 90–120 minutes, focusing on proprioceptive and functional training exercises.<br>- 40 to 64 supervised sessions over the initial 8 months post-surgery.

Resources:

1. Schenck RC, Blaschak MJ, Lance ED, Turturro TC, Holmes CF. A prospective outcome study of rehabilitation programs and anterior cruciate ligament reconstruction. Arthrosc J Arthrosc Relat Surg Off Publ Arthrosc Assoc N Am Int Arthrosc Assoc. 1997 Jun;13(3):285–90.

2. Beard DJ, Dodd CA. Home or supervised rehabilitation following anterior cruciate ligament reconstruction: a randomized controlled trial. J Orthop Sports Phys Ther. 1998 Feb;27(2):134–43.

3. Fischer DA, Tewes DP, Boyd JL, Smith JP, Quick DC. Home based rehabilitation for anterior cruciate ligament reconstruction. Clin Orthop. 1998 Feb;(347):194–9.

4. Grant JA, Mohtadi NGH, Maitland ME, Zernicke RF. Comparison of home versus physical therapy-supervised rehabilitation programs after anterior cruciate ligament reconstruction: a randomized clinical trial. Am J Sports Med. 2005 Sep;33(9):1288–97.

5. Ugutmen E, Ozkan K, Kilincoglu V, Ozkan FU, Toker S, Eceviz E, et al. Anterior cruciate ligament reconstruction by using otogenous [correction of otogeneous] hamstring tendons with home-based rehabilitation. J Int Med Res. 2008;36(2):253–9.

6. Revenäs Å, Johansson A, Leppert J. A randomized study of two physiotherapeutic approaches after knee ligament reconstruction. Adv Physiother. 2009 Jan 1;11(1):30–41.

7. Grant JA, Mohtadi NGH. Two- to 4-year follow-up to a comparison of home versus physical therapy-supervised rehabilitation programs after anterior cruciate ligament reconstruction. Am J Sports Med. 2010 Jul;38(7):1389–94.

8. Hohmann E, Tetsworth K, Bryant A. Physiotherapy-guided versus home-based, unsupervised rehabilitation in isolated anterior cruciate injuries following surgical reconstruction. Knee Surg Sports Traumatol Arthrosc Off J ESSKA. 2011 Jul;19(7):1158–67.

9. Przybylak K, Sibiński M, Domżalski M, Kwapisz A, Momaya AM, Zielińska M. Supervised physiotherapy leads to a better return to physical activity after anterior cruciate ligament reconstruction. J Sports Med Phys Fitness. 2019 Sep;59(9):1551–7.

10. Lim JM, Cho JJ, Kim TY, Yoon BC. Isokinetic knee strength and proprioception before and after anterior cruciate ligament reconstruction: A comparison between home-based and supervised rehabilitation. J Back Musculoskelet Rehabil. 2019;32(3):421–9.

11. Rhim HC, Lee JH, Lee SJ, Jeon JS, Kim G, Lee KY, et al. Supervised Rehabilitation May Lead to Better Outcome than Home-Based Rehabilitation Up to 1 Year after Anterior Cruciate Ligament Reconstruction. Med Kaunas Lith. 2020 Dec 28;57(1):19.

12. Syed RIB, Hangody LR, Frischmann G, Kós P, Kopper B, Berkes I. Comparative Effectiveness of Supervised and Home-Based Rehabilitation after Anterior Cruciate Ligament Reconstruction in Competitive Athletes. J Clin Med. 2024 Apr 12;13(8):2245.
